# Microphysiological systems as a pillar of the Human Exposome Project

**DOI:** 10.1016/j.jbc.2025.110782

**Published:** 2025-10-04

**Authors:** Fenna C.M. Sillé, Lena Smirnova, Thomas Hartung

**Affiliations:** 1 Center for Alternatives to Animal Testing (CAAT), Department of Environmental Health Sciences, Johns Hopkins Bloomberg School of Public Health and Whiting School of Engineering, Johns Hopkins University, Baltimore, Maryland, USA; 2 Doerenkamp-Zbinden-Chair for Evidence-based Toxicology, Department of Environmental Health Sciences, Johns Hopkins Bloomberg School of Public Health and Whiting School of Engineering, Johns Hopkins University, Baltimore, Maryland, USA; 3 CAAT-Europe, University of Konstanz, Konstanz, Germany; 4 CAATevents, Solingen, Germany

**Keywords:** microphysiological systems, organ-on-chip, human exposome, toxicology, risk assessment, new-approach methodologies

## Abstract

The Human Exposome Project (HEP) aims to decode how lifelong environmental exposures shape health and disease, complementing genomic insights with a systems-level understanding of external influences. Achieving this vision requires experimental platforms that move beyond the limitations of animal models, which often lack human relevance and mechanistic resolution. Microphysiological systems (MPSs)—including organoids and organs-on-chips derived from human stem cells—offer such an opportunity. These engineered models recapitulate human tissue architecture and function under controlled conditions, enabling direct study of exposure–response relationships at the cellular and organ levels. In this review, we outline how MPS can serve as a foundation for exposome research by bridging epidemiological observations with mechanistic biology. We describe applications ranging from air pollutant toxicity to food contaminants, endocrine disruptors, and nanomaterials, highlighting how MPS integrated with omics technologies and artificial intelligence can reveal pathways of injury, identify biomarkers, and support the development of digital twins to simulate exposure–disease trajectories. We also discuss frameworks for validation, quality assurance, and transparent reporting, which are essential for reproducibility and regulatory acceptance. Finally, we consider ethical issues, such as donor rights, data sovereignty, and equitable access, underscoring the importance of anticipatory governance. Together, MPS represent more than alternatives to animal testing—they are strategic enablers of a human-relevant, artificial intelligence–empowered exposome science. By anchoring statistical associations in mechanistic data, MPS can accelerate translation into public health policies that are predictive, preventive, and personalized.

The exposome, defined as the totality of environmental exposures an individual encounters from conception onward (1, 2), is increasingly recognized as a critical determinant of human health. Unlike the relatively static genome, the exposome is dynamic, complex, and shaped by external chemical, physical, biological, and social factors throughout life. Understanding the exposome’s role offers the potential to transform biomedical research by complementing genomic insights with a more complete picture of disease etiology and progression. Traditional toxicology—built primarily on animal models—has contributed foundational insights but is increasingly strained by ethical, logistical, and scientific limitations. Animal studies are resource intensive, slow, and often fail to reliably predict human responses. High-profile examples from the poor translation of preclinical efficacy data underscore the translational gap between animal models and human health outcomes (3, 4). Systematic reviews and meta-analyses reveal that animal-based approaches suffer from low reproducibility, species differences, and inconsistent methodology, undermining their predictive value in the human context (5). Notably, the US Food and Drug Administration (FDA) and US Environmental Protection Agency have, in recognition of this problem, started initiatives to transition with support by the National Institutes of Health to human-relevant new approach methods (6).

Microphysiological systems (MPSs), an umbrella term coined by the FDA for organoid and organs-on-chip systems often based on stem cell technologies (Fig. 1), are emerging as key tools to bridge this gap. These bioengineered *in vitro* models replicate human tissue architecture and function, offering a more accurate and ethical alternative to animal testing. When integrated with omics data and exposome analytics, MPSs offer an unprecedented opportunity to study mechanistic exposure–disease relationships under controlled, human-relevant conditions. As such, MPSs are not only a technological innovation but a strategic pillar in realizing the vision of a Human Exposome Project (HEP) (https://basicresearch.defense.gov/Portals/61/Documents/future-directions/) (7)—a coordinated effort to decode the environmental determinants of health with the same ambition and transformative potential as the Human Genome Project.

**Figure 1:**
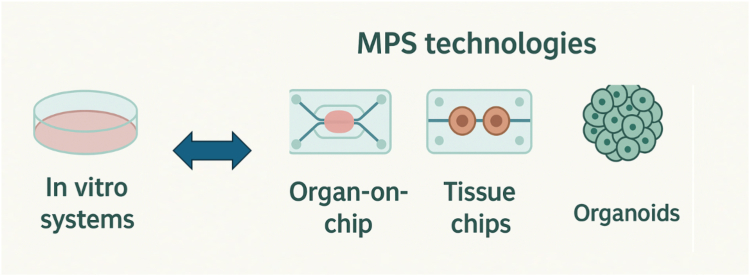
**Traditional cell culture *versus* MPS technologies.** The diagram shows the most prominent MPS technologies, that is, 3D cultures such as organoids, single-organ-on-chip, and multiorgan-tissue-chips, aka human-on-chip or patient-on-chip. MPS, microphysiological system.

## Evolution and capabilities of MPS

MPSs, encompassing organ-on-chip and organoid technologies, have evolved rapidly over the past decade. Initially developed through advances in microsystems engineering and cell biology, these platforms recreate key physiological features, such as 3D architecture, tissue–tissue interfaces, fluid flow, and mechanical cues that are critical for mimicking human organ functions (8).  Historically, the concept of MPS was framed around dynamic microfluidic systems involving perfused tissue compartments. The National Institutes of Health/National Center for Advancing Translational Sciences Tissue Chip program, launched in 2012, and our first transatlantic think tank for toxicology (t^4^) workshop in 2015 (9) laid the groundwork for a 25-year roadmap toward "you-on-a-chip" models for disease modeling and drug development (10, 11). The field has since expanded to include static and semidynamic organoids, membrane-based coculture models, and bioprinted tissues—now collectively recognized as part of the MPS spectrum (10).  Single-organ MPS models have become increasingly sophisticated, incorporating features, such as vascularization, immune competence, and sensor integration. The combination of induced pluripotent stem cell (iPSC)–derived cell types and real-time biosensors allows for dynamic monitoring of physiological responses and enables disease modeling under patient-specific conditions (11). Multiorgan systems have also matured, with several platforms integrating up to four autologous tissues, although challenges remain in achieving full-body physiology, in some proof-of-principle studies where even 10 to 12 organ equivalents were perfused for a month (12, 13).

The stakeholder landscape has broadened considerably. Academic laboratories drive innovation and disease modeling; the MPS supplier industry focuses on standardization and platform scalability; end-user industries, particularly pharma and biotech, apply MPS for early drug discovery and toxicology; and regulators are increasingly engaging in qualification and harmonization efforts. The establishment of the International MPS Society (iMPSS) (https://impss.org) and the MPS World Summit (https://mpsworldsummit.org) series has catalyzed global communication, standard setting, and strategic planning across sectors.  Recent technological advances (14) include the integration of advanced imaging, ∼omics, and biosensor modalities for richer data capture, along with efforts toward automated, artificial intelligence (AI)–assisted readouts. These developments enable more predictive and reproducible MPS-based assays and support the long-term vision of digital MPS twins (aka AI-calibrated virtual counterparts of real human patients based on MPS and omics analyses) for public health and precision medicine applications.

## Applications of MPS in exposure science

MPSs are rapidly gaining prominence in exposure science as powerful, human-relevant platforms to elucidate how environmental agents influence biological processes (15). Their ability to replicate tissue- and organ-level physiology *in vitro* positions them uniquely to assess complex, real-world exposures (Table 1). MPSs have been employed to model a broad range of environmental exposures (16, 17), including air pollutants (*e.g.*, PM2.5, ozone) (18, 19), food additives (20, 21), endocrine-disrupting chemicals (22), and nanomaterials (23, 24, 25). For example, lung-on-chip systems replicate breathing mechanics to study inhalation exposures (26), whereas gut–liver (27) and skin–liver platforms (28) enable the assessment of oral and dermal exposures and associated metabolism. These models permit continuous perfusion and multiroute simulation of chemical exposures, reflecting real-life complexity far better than traditional systems.

**Table 1:** Comparing the promise of MPS in an exposome paradigm *versus* traditional toxicology

Aspect	MPS + exposome	Traditional toxicology
Human relevance	High—based on human cells/tissues	Low —based on animal models
Human predictivity	High—human-relevant responses	Low to moderate—poor translation
Mechanistic insight	Strong—mechanistic data generation	Limited—often apical endpoints
Throughput	Moderate—increasing with automation	Low—based on animal models
Scalability	Moderate—scalable with investment	Low—based on animal models
Ethical considerations	High—nonanimal, ethically preferred	Low—animal welfare concerns
Cost per data point	Moderate—decreasing with miniaturization	High—because of animal housing/testing
Integration with omics/AI	Strong—designed for integration	Weak—typically not integrated

The table summarizes the expected increase in relevance, throughput, and scalability of the new over the traditional approach.

MPS-based models have proven particularly valuable in neurodevelopmental toxicity studies (14). Brain-on-chip platforms and blood-brain barrier MPSs have revealed developmental disruptions and impaired barrier function after pesticide and heavy metal exposure, supporting early-life exposure risk assessment (29, 30, 31, 32). Similarly, liver-pancreatic islet MPSs have modeled metabolic dysfunction from dietary exposures (33, 34), whereas immune-competent MPSs are shedding light on inflammation and immunotoxicity mechanisms (35). Disease-specific MPS platforms have also been established for metabolic, cardiovascular, and immunological endpoints, enabling MicroPathoPhysiological Systems (36).

The integration of MPS with ∼omics technologies, particularly metabolomics, transcriptomics, and proteomics, has transformed exposure science from observational to mechanistic. High-resolution mass spectrometry (37) combined with MPSs enables real-time tracking of cellular responses, metabolic pathway perturbations, and adaptive stress responses to toxicants. Notably, untargeted metabolomics offers vast potential for exposome-wide profiling, although structural identification remains a key bottleneck. Advances in AI-enabled retention time prediction and quantitative structure–activity relationship modeling–based annotation frameworks are addressing this challenge and improving chemical identification confidence (38).  By coupling physiologically relevant human models with high-content omics and machine learning analytics, MPS-based exposure platforms are poised to bridge the critical gap between environmental measurements and health outcomes. These systems can support causal inference, risk stratification, and biomarker discovery by AI (39), helping realize the HEP’s vision of proactive, exposure-informed health protection.

MPS technologies are increasingly employed in exposure science to model the effects of environmental agents under controlled, human-relevant conditions. They provide a platform to assess both acute and chronic responses to complex mixtures and low-dose exposures, bridging the translational gap between epidemiological data and mechanistic understanding. MPS integration with ∼omics technologies, particularly transcriptomics, metabolomics, and proteomics, enables high-dimensional readouts. These platforms facilitate the discovery of biomarkers (40, 41, 42), adverse outcome pathways, and molecular signatures of toxicity. Combined with machine learning, MPS-omics datasets can identify causal links between exposures and disease phenotypes, making them ideal for exposomics studies.

Mechanistic insights gained from MPS applications are increasingly valuable for exposure science. For example, lung-on-chip models not only simulate breathing mechanics but also reveal oxidative stress, tight-junction disruption, and proinflammatory cytokine release after particulate or oxidant exposures, recapitulating early hallmarks of airway injury (26, 43, 44, 45). Similarly, gut–liver axis chips demonstrate how food contaminants and microbiome metabolites perturb bile acid secretion and xenobiotic metabolism—mechanistic endpoints difficult to capture in static assays (46, 47, 48). Brain organoids and blood-brain barrier MPS reveal that pesticide exposures can impair neurite outgrowth, synaptic signaling, and barrier integrity, anchoring neurodevelopmental toxicity to specific cellular pathways (30, 49, 50). Immune-competent MPSs have elucidated how nanomaterials activate NLRP3 inflammasome signaling and alter cytokine dynamics (51, 52). When combined with omics approaches, these readouts map to adverse outcome pathways (53), enable biomarker discovery, and support causal linkage between exposure and disease phenotypes—moving exposure science from correlative associations to human-relevant mechanisms.

Overall, MPSs are poised to become indispensable tools for mechanistic toxicology and exposome research. They offer human-relevant, ethically preferable alternatives to animal models and enable personalized and population-level risk assessments when combined with exposomic data, ∼omics technologies, and AI-powered analytics.

## MPS and the HEP

**Figure 2:**
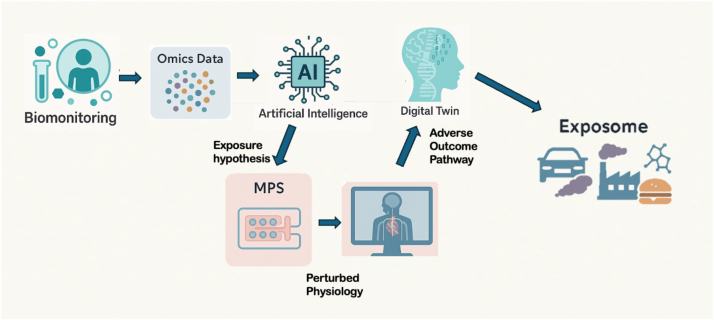
**MPS in the context of the exposome.** This workflow shows how human samples, especially biofluids, such as blood and urine, analyzed with ∼omics and artificial intelligence, deliver an exposure hypothesis. MPSs serve to test this hypothesis in a human-relevant tissue model. Mapping these effects on the perturbed physiology and biochemistry enables a systems biology approach and ultimately digital twins. By modeling populations of such biological twins representing human diversity, the human exposome builds as the integrated actionable knowledge of the exposure side of disease. MPS, microphysiological system.

**Figure 3:**
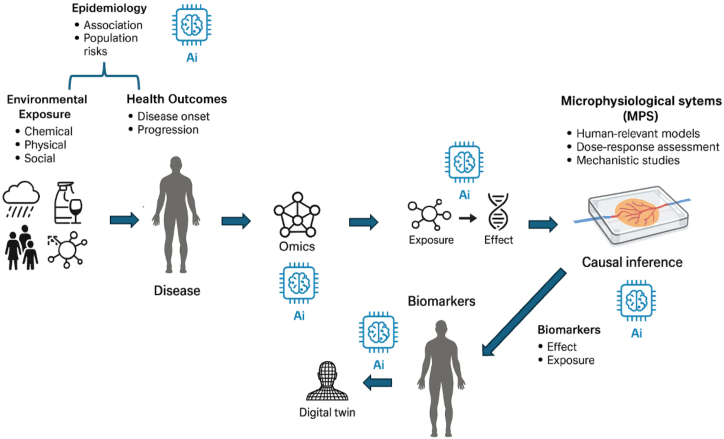
**How AI enables a human exposome?** AI supports multiple steps of the exposome: First, it supports the more traditional epidemiological approach of identifying exposure—disease relationships. Second, it makes sense of (multi-)omics measurements of biomonitoring samples, ultimately deducing hypotheses of exposure–effect relationships. When testing these hypotheses in MPS, AI can support the deduction of biomarkers of possible clinical relevance. These can be tested in human populations and expanded with AI to the digital twin modeling approach. AI, artificial intelligence; MPS, microphysiological system.

MPSs are central to the realization of the HEP, providing a human-relevant, mechanistic platform to link environmental exposures with health outcomes (Fig. 2). Their ability to reproduce complex physiological processes *in vitro* makes them invaluable for validating exposure biomarkers, deciphering causal mechanisms, and bridging observational exposomic data with experimental evidence.  The synergy between MPS, biomonitoring, untargeted metabolomics, and AI enables an integrative exposome framework. Biomonitoring offers snapshots of internal chemical burdens, whereas MPS allows for hypothesis-driven testing of these exposures under controlled conditions. Omics-enhanced MPS can recapitulate tissue-specific responses to measured blood levels of xenobiotics, generating mechanistic data that complement epidemiological findings. AI plays a pivotal role in this context of forming an exposure to the adverse outcome hypothesis. MPS then provides a tractable system for testing causal hypotheses generated from exposomics and biomonitoring studies. When individuals show differential susceptibility to environmental factors, MPS—especially when derived from iPSCs of exposed individuals—can recapitulate individual-specific responses. Human iPSC–derived cardiomyocytes integrated into cardiac organ-on-chip platforms exhibit oxidative stress, altered Ca^2+^ handling, and contractile dysfunction following exposure to cigarette or e-cigarette smoke extracts, and documented interdonor variability in iPSC-cardiomyocyte phenotypes suggests that exposure-stratified studies (*e.g.*, smokers *versus* never-smokers) are feasible and likely to reveal differential susceptibility (54). This makes MPS an essential platform for functional follow-up of statistical associations and for derisking targets in public health interventions. The convergence of *in vitro* MPS and *in silico* modeling heralds a new era of digital twins (55, 56, 57, 58) for toxicology and public health (Fig. 3).

In the future, digital MPS twins—virtual models trained on biological MPS and other data—enable simulation of exposure scenarios, dose extrapolation, and predictive analytics across populations (59). This dual biological–computational pipeline facilitates longitudinal inference, supports evidence-to-decision (EtD) frameworks, and reduces reliance on animal testing. In this integrated ecosystem, MPSs are not merely tools but act as foundational infrastructure for exposome science. Their deployment within AI-powered, omics-informed frameworks enables the mechanistic anchoring of exposomic associations and establishes a pathway toward individualized and preventive health strategies informed by environmental risk.

## Validation, qualification, and quality assurance

The increasing adoption of MPS in research and safety assessment demands robust processes to ensure model reliability, reproducibility, and regulatory acceptance. A foundational distinction must be made between validation and qualification. Validation typically refers to a comprehensive demonstration of test reliability and relevance—often benchmarked against animal models—while qualification is more flexible and context specific, emphasizing the model’s fitness for purpose in a defined application space (42). To support robust method development, Good Cell and tissue Culture Practice (GCCP 2.0) offers a comprehensive framework tailored to modern cell and tissue culture systems, including stem cells and MPSs (60, 61). It emphasizes standardization, traceability, education, and quantification of physiological function to enhance reproducibility and minimize interlab variability. Building on this, the e-validation concept modernizes validation by introducing AI-based, dynamic, and transparent performance benchmarking (62). Rather than anchoring to fixed reference datasets or legacy animal data, e-validation enables continuous model refinement, contextual evidence accumulation, and alignment with human biology to implement validation based on mechanistic validation (63). This is particularly suited to complex, evolving platforms like MPS.

Equally important is rigorous reporting. The Good In Vitro Reporting Standards (GIVReSt) initiative (64, 65) directly targets the reproducibility crisis in *in vitro* science by defining six key principles for transparency in reporting. These range from complete documentation of cell identity and quality control to detailed accounts of culture protocols, endpoint assays, and data handling. GIVReSt complements GCCP by ensuring that published methods are sufficiently detailed for independent replication, that is, a detailed reporting framework designed to improve transparency, reproducibility, and traceability in *in vitro* research, especially for complex models like MPS. GIVReSt builds on six key principles covering model documentation, procedural transparency, and data accessibility, drawing inspiration from initiatives like MIAME (https://www.ncbi.nlm.nih.gov/geo/info/MIAME.html) (Minimum Information About a Microarray Experiment), CONSORT (https://www.equator-network.org/reporting-guidelines/consort/) (guideline for reporting randomized trials), and ARRIVE (https://arriveguidelines.org) (Animal Research: Reporting of In Vivo Experiments). Critically, GIVReSt is designed to be interoperable with FAIR data (https://www.go-fair.org/fair-principles/) (Guiding Principles for scientific data management and stewardship) principles and supports the use of AI-based tools to automate compliance checks, enhance peer review, and streamline evidence synthesis. This dual strategy—of quality execution (GCCP) and transparent communication (GIVReSt)—offers a robust foundation for confidence in MPS-based safety science, particularly as such models enter regulatory evaluation and support causality assessment in the HEP.

Together, qualification frameworks (42), GCCP 2.0 (48, 49), fit-for-purpose criteria to establish quality management (66), Next generation validation (67), e-validation (50), risk-of-bias assessment (68), and GIVReSt (52, 54) provide a powerful quality assurance ecosystem. Together, these advances offer a path toward fit-for-purpose validation of MPS as next-generation safety tools. They enable evidence-based confidence (69) in MPS-generated data, support regulatory readiness, and accelerate the acceptance of human-relevant models in toxicology and exposure science.

## Challenges and solutions

Despite their transformative potential, MPS technologies face several challenges that must be addressed for widespread adoption in exposure science and regulatory contexts. These include the lack of standardization, limited throughput, high cost, and the technical complexity of designing and operating multiorgan systems. Standardization remains a critical bottleneck. The diversity of platforms, cell sources, culture formats, and readouts complicates interlaboratory reproducibility and comparability. Without consistent standards for model qualification and performance criteria, benchmarking across platforms is difficult. Initiatives like the iMPSS (https://impss.org) are actively developing standards for reporting, performance, and interoperability to address these gaps, whereas the Center for Alternatives to Animal Testing (https://caat.jhsph.edu) (CAAT)–coordinated GCCP 2.0 and GIVReSt frameworks provide foundations for method consistency and transparency.

Throughput and cost present practical limitations. MPS platforms, particularly those incorporating microfluidics or multiorgan integration, are often resource intensive and low throughput, limiting their application in large-scale screening. Proposed solutions include modular designs that allow stepwise complexity, for example, as a plug-and-play platform, automation and robotics for parallelization, and AI-driven analytics to reduce manual interpretation.

Another challenge lies in defining the applicability domain and context of use (CoU) for each MPS model. Regulatory confidence hinges on clarity around what a model is designed to predict and under what conditions it performs reliably. Efforts by iMPSS, CAAT, and regulators are advancing CoU frameworks, emphasizing early engagement with end users and fit-for-purpose validation strategies. To address these collective barriers, CAAT and iMPSS have proposed a roadmap for ecosystem development (11, 14). This includes establishing reference models for key tissues, investing in open-source protocols and standard operating procedures, engaging stakeholders in cocreation, and aligning efforts with international regulatory harmonization bodies. Central to this vision is a federated innovation model—where academia, startups, industry, and regulators codevelop tools and benchmarks to ensure translational impact and sustainability. By acknowledging and systematically addressing these challenges, the MPS field can evolve from a promising innovation space to a robust pillar of 21^st^-century human health science.

## Toward implementation: from new approach methods to public health

The ultimate promise of MPS technologies lies in their integration into decision-making frameworks that guide regulatory policy and public health interventions (70). This transition requires not only scientific validation but also acceptance by stakeholders who translate mechanistic findings into societal action. EtD frameworks provide a structured path to achieve this, combining diverse data sources, expert judgment, and defined criteria for risk assessment, clinical guidelines, and evidence-based approaches. EtD frameworks are structured tools designed to transparently guide how scientific evidence—such as data from MPS, epidemiology, or omics—is translated into actionable decisions. They support regulatory and clinical processes by integrating diverse data types with expert judgment, stakeholder values, and predefined criteria, such as benefits, harms, feasibility, and equity. In doing so, EtD frameworks promote consistency, transparency, and reproducibility in forming public health guidelines, risk assessments, and policy decisions.

The IMPACT program (7) represents a cornerstone in this translational vision. IMPACT stands for *Implementation Moonshot Project for Alternative Chemical Testing* (7) but might as well be spelled out as “*Integrating Microphysiological Platforms And Computational Tools*.” Spearheaded by CAAT and supported by a growing global consortium, IMPACT seeks to operationalize an HEP by combining MPS, ∼omics, AI, and real-world exposure data. The recent organization of the Exposome Moonshot Forum (https://exposomemoonshot.org) (71) (https://factor.niehs.nih.gov/2025/6/feature/global-exposome-consortium) brought together ∼300 global eminent researchers and representatives of the civil society to discuss the translation of exposomics research to real-life applications for the HEP (72) (https://www.niehs.nih.gov/news/factor/2025/6/feature/global-exposome-consortium). The program was structured around use-case pipelines that span early exposure modeling, mechanistic pathway elucidation, and application in regulatory and clinical contexts. These pipelines fuel the shaping of HEP. A critical enabler of this transition is cross-sector collaboration. Public–private partnerships are essential for building scalable infrastructure, harmonizing regulatory frameworks, and sharing data and best practices. The Exposome Moonshot Forum convened key actors from government agencies, academia, industry, and nonprofits to chart a unified roadmap for implementation. Topics included coordinated investment, precompetitive consortia, and alignment with international initiatives like the *Network for EXposomics in the United States* (NEXUS) (https://www.nexus-exposomics.org), the *Human Exposome Assessment Platform* (HEAP) https://heap-exposome.eu, *European Human Exposome Network* (EHEN) (https://www.humanexposome.eu), now transformed into the International Human Exposome Network (IHEN) (https://humanexposome.net) and the *Research Infrastructure for EnvIRonmental Exposure assessmeNt in Europe* (EIRENE) (https://eirene.eu) in Europe. Taken together, these efforts reflect a fundamental shift—from reactive safety assessments to proactive health protection guided by exposure science. MPS, as core enablers of this shift, will help deliver a future where environmental health policies are informed by human biology, supported by predictive technologies, and cocreated by a diverse community of stakeholders. The Moonshot Forum ended with the *Washington Declaration on the Human Exposome—Establishment of a Global Consortium* (https://exposomemoonshot.org/washington-d-c-declaration-on-the-human-exposome/). The next meeting of the Global Exposome Forum (https://globalexposomeforum.org) now created will take place in Barcelona in April 2026.

## Ethical foundations for MPS and the HEP

As the HEP evolves into a transformative infrastructure for preventive health, the ethical dimensions of MPS and exposome research must be recognized not as peripheral concerns but as foundational pillars. The convergence of human-derived biomaterials, complex *in vitro* systems, continuous environmental monitoring, and AI-based analytics presents novel ethical challenges that exceed traditional biomedical frameworks. In this chapter, we outline a multilayered ethical landscape anchored in three interdependent domains: bioengineering ethics, exposome ethics, and regulatory risk ethics.

### Bioengineering ethics: from donor rights to moral consideration of constructs

MPSs, especially organoids and body-on-a-chip systems, raise important questions of consent, ownership, and potential moral status. As Gaillard (73) argues in a perspective article, the life cycle of these constructs often extends far beyond the scope foreseen by cell donors or even researchers. Static, one-time informed consent may be inadequate in this context. Instead, dynamic consent and governance-based models—where donor representatives participate in decision making—should guide the use of human biomaterials, particularly for long-lived, repurposable cell lines and chimeric constructs.

Furthermore, ethical deliberation must anticipate future scenarios in which complex MPS, particularly neural or embryo-like constructs, might be viewed as possessing moral status because of functional integration, sentience (74), or self-organization. While speculative, such developments call for proactive oversight, similar to evolving frameworks in synthetic biology and brain organoid research.

### Exposome ethics: participatory justice and data sovereignty

HEP will generate vast, multimodal data streams linking biospecimens, environmental exposure histories, behavior, and socioeconomic status. The ethical implications of such high-resolution, longitudinal monitoring are profound. The Sillé *et al.* (2025) exposome ethics framework (75) identifies five central domains: privacy and data sovereignty, informed consent and engagement, environmental justice, governance and oversight, and actionability of findings.

Data generated from MPS-integrated exposomics—such as organoid responses to pollutant mixtures or AI-driven exposure predictions—must respect community rights to data control and benefit. This includes ensuring algorithmic fairness, transparency in model development, and the contextual interpretation of findings to avoid stigmatization or inequitable interventions. Justice in exposomics is not merely distributive, but procedural, requiring the institutionalization of participatory structures such as community advisory boards and a proposed Exposome Ethics Consortium.

### Regulatory risk ethics: decision making under uncertainty

The integration of MPS into regulatory science introduces ethical tensions between innovation, precaution, and societal accountability. Bhuller *et al.* (76) proposed a "projector model" of ethical risk governance that emphasizes 10 principles, including minimizing harm, maintaining trust, reducing disparities, and promoting transparency and fairness. These principles align closely with the goals of MPS-based risk assessment and should be explicitly integrated into validation, qualification, and EtD processes.

Especially relevant is the idea of validation ethics (56): the shift from rigid animal concordance benchmarks toward probabilistic, human-relevant validation pathways that foreground transparency, uncertainty communication, and relevance to exposed populations (67). In the era of next-generation testing strategies, ethical validation becomes both a scientific and societal imperative.

## Strategic outlook and remaining barriers

The emergence of MPS marks a paradigm shift in our approach to understanding and mitigating the health effects of environmental exposures. These technologies enable a transition from traditional animal-based toxicology to a postanimal era grounded in human biology, mechanistic insight, and systems-level analysis. With their capacity to replicate human tissue function and integrate with ∼omics, biosensors, and AI, MPSs are not merely alternative tools—they are foundational to a mechanism-based understanding of the exposure contribution to disease. Looking ahead, MPSs stand as cornerstone technologies for a data-driven, AI-enhanced exposome future. Their use in conjunction with high-throughput omics and machine learning offers unprecedented granularity in modeling complex exposure–health relationships. The concept of a digital twin—combining physical MPS platforms with computational surrogates—promises to revolutionize personalized risk assessment, predictive toxicology, and precision public health. Realizing this vision will require bold action. The scientific community must invest in transdisciplinary research that links exposure science, bioengineering, computational modeling, and epidemiology. Regulators must adapt frameworks to embrace evidence from MPS (https://policylabs.frontiersin.org/content/commentary-roadmap-to-reduce-animal-testing) (6) and ensure that fit-for-purpose qualification replaces legacy validation constraints. Industry and public agencies must jointly support public–private consortia, infrastructure development, and training programs to build a skilled workforce capable of leveraging these tools. The HEP, anchored in MPS and enabled by exposomics and AI, offers a once-in-a-generation opportunity to reimagine environmental health science. By aligning innovation, evidence, and policy, we can create a future where risk assessments are not only human relevant but anticipatory—protecting health before harm occurs.

Despite the extraordinary progress in the development, application, and regulatory interest in MPS, substantial challenges remain before these technologies can realize their transformative potential as cornerstones of an HEP. This final chapter outlines the strategic roadblocks and the opportunities they present.

### Overcoming the complexity barrier

The biological complexity and engineering sophistication of MPS are both their greatest strengths and core challenges. Integrating multiple cell types, achieving physiological scaling across tissues, incorporating immune components, and maintaining long-term functionality all require not just technical excellence but robust quality management systems. Efforts such as GCCP 2.0 and fit-for-purpose quality frameworks are advancing, but greater harmonization is needed across laboratories, particularly for multiorgan or vascularized systems. Automation, bioprinting, and closed-loop monitoring using integrated sensors and AI will be essential to scale these technologies for routine use.

### Standardization without stagnation

Lack of standardized protocols, terminology, and performance metrics continues to fragment the field. There is an urgent need for community-driven standards that do not constrain innovation but create a baseline of comparability, much like GIVReSt aims for in reporting practices. A future global consortium under the umbrella of organizations like the iMPSS could serve as the steward for these standards, ensuring consensus-based evolution rather than regulatory rigidity.

### Validation: from binary to fit for purpose

The traditional binary paradigm of "*validated vs. not validated*" is ill suited for the evolving and application-specific nature of MPS. Instead, a shift toward "readiness levels" (77) and tiered, CoU qualification pathways is essential. AI-driven e-validation (50, 78) approaches, biomarker-based mechanistic validation (42), and cloud-based reference libraries of compounds and responses could streamline this process while promoting transparency and comparability.

### Regulatory integration and trust building

Uncertainty around regulatory expectations remains a deterrent for many developers. The path forward includes publishing successful case studies, expanding participation in regulatory sandbox initiatives, and establishing international working groups to codevelop guidelines with agencies like FDA, EMA, and Organization for Economic Co-Operation and Development. Acceptance of MPS for certain regulatory decisions—such as prioritization, screening, and early efficacy—can pave the way for broader adoption in hazard and risk assessment.

### Socioeconomic accessibility and global equity

As MPS technologies mature, their affordability and accessibility must be addressed. Academic labs, low-resource regions, and public health institutions must be able to engage with MPS, particularly as part of a global HEP. To ensure equitable access to MPS technologies, particularly in low- and middle-income countries, open-source infrastructure must be prioritized. Proprietary platforms, high licensing costs, and limited access to validated models pose significant barriers to participation. Shared resources—such as open-source chip designs, public biobanks of diverse iPSCs, distributed training hubs, community-curated validation datasets, and digital twins that reduce experimental burden could democratize access. Harmonized protocols and open-access digital twins trained on real-world exposure data are essential to support inclusive, globally relevant exposome science. Without deliberate investment in infrastructure that supports distributed innovation, there is a risk that the benefits of MPS-enabled exposomics will remain confined to well-resourced settings—further entrenching, rather than alleviating, global health disparities.

### Toward a convergent future

The convergence of MPS with AI, exposomics, and personalized medicine is not merely additive—it is multiplicative. MPS-based digital twins can simulate individual responses to environmental exposures, enabling rapid hypothesis testing, guiding longitudinal cohort studies, and supporting precision public health interventions. Such a vision requires not only technical innovation but new models of collaboration, governance, and data ethics.

## Conflict of interest

T. H. is named inventor on a patent by Johns Hopkins University on the production of minibrains (also called BrainSpheres or brain organoids), which is licensed to Axo-Sim, recently renamed 28Bio, New Orleans, LA, USA. T. H. and L. S. are consultants for 28Bio, New Orleans, and T. H. is also a consultant for the American Type Culture Collection, Manassas, VA, InSphero, Zurich, Switzerland, Crown Biotech, San Diego, CA, and was until recently for AstraZeneca on advanced cell culture methods.
